# Learning from Nigeria’s public-private pathways to generate inclusive healthcare data

**DOI:** 10.1038/s43856-026-01984-x

**Published:** 2026-10-21

**Authors:** Yash Raj Shrestha, Zeynep Erden, Öykü Isik, Katharina Demmel, Spyros Tsevas, Olatoke Oluwadara, Sam McAvoy, Johan Descombes, Alma Rubio

**Affiliations:** 1 University of Lausanne, Lausanne, Switzerland; 2 Zurich University of Applied Sciences, Zurich, Switzerland; 3 Arizona State University, Tempe, AZ USA; 4 F. Hoffmann-La Roche AG, Basel, Switzerland; 5 Genentech, Inc. 1 DNA Way, South San Francisco, CA USA

**Keywords:** Public health, Epidemiology

## Introduction

Artificial Intelligence (AI) is transforming healthcare, with applications spanning disease diagnosis, patient monitoring, personalized treatments, and drug discovery^1,2^. There is growing consensus among healthcare providers, policymakers, and patients that AI-enabled digital health is reshaping care delivery by accelerating diagnoses, improving outcomes, reducing costs, and expanding access to remote regions. Yet, despite these advances, a persistent digital divide threatens to widen health inequities. The datasets powering AI systems remain predominantly sourced from white, Western populations, leaving racial and ethnic minorities and entire regions such as Africa underrepresented^3^. This lack of data diversity produces AI models that are less accurate, less generalizable, and potentially biased, exacerbating disparities in healthcare outcomes^4^. In addition, African Americans are less likely to participate in health research due to longstanding mistrust, as seen in HIV, cancer prevention, and occupational health studies^5,6^. The consequence is a problematic cycle with underrepresentation in data leading to underperformance in care delivery^7^.

Bridging this data divide is not merely a technical challenge, but also a moral and public health imperative. Equitable AI-enabled healthcare requires amplification of data from underrepresented populations, especially from Africa. While Africa faces significant obstacles to data collection, including fragmented infrastructure, financial barriers, and decentralized systems, it also presents an unparalleled opportunity for innovation. By strengthening data ecosystems and fostering inclusive public-private partnerships, African nations can advance toward equitable, AI-powered care.

This commentary focuses on Nigeria, a nation with systemic healthcare challenges but also a setting for a range of collaborations between government, pharmaceutical companies, research institutions, and non-governmental organizations (NGOs). Drawing from our multi-year collaboration involving universities in Switzerland, Roche Pharmaceuticals, Genentech, and healthcare stakeholders in Nigeria, we highlight how public-private partnerships can overcome data scarcity and build sustainable, inclusive health data infrastructures. We have years of experience in the pharmaceutical industry, have undertaken extensive fieldwork in Nigeria, and have interviewed numerous healthcare providers, government officials, patient advocacy groups, and pharmaceutical companies. We argue that Nigeria’s experience offers a set of transferable design principles rather than a ready-made model, and we specify below the contextual conditions and governance safeguards on which their transfer depends.

## The healthcare system in Nigeria

Healthcare faces significant challenges in Nigeria. Financial constraints severely limit access to healthcare facilities, leaving a substantial portion of the population uninsured and unable to afford essential medical services. With <10% of the population having health insurance coverage^8^, the government operates a federal insurance system supplemented by state and private plans. The healthcare system is complex and lacks centralized electronic health records (EHR) and decentralized data systems, hindering effective healthcare delivery. Consequently, data collection is skewed towards urban areas, excluding those who rely on out-of-pocket care. Despite initial efforts, uniform data collection systems are not yet in place, complicating comprehensive healthcare analysis and the ability to address needs.

These challenges highlight the urgent need for comprehensive reforms and investments in healthcare infrastructure and data systems to ensure equitable access. Despite these obstacles, there have been notable public-private collaborations that are driving progress and can inspire other African and developing countries.

## Collaborative efforts to improve health data access in Nigeria

In resource-constrained settings, partnerships are often the most practical route to improving access and generating usable health data, provided that incentives and accountability are explicit. Below, we highlight Nigerian examples that illustrate workable designs.

### Pharmaceutical-government partnership

To improve access to treatments for insured individuals, the Nigerian government has partnered with pharmaceutical firms, including Roche, under the National Health Insurance Agency (NHIA). This collaboration has given rise to a co-payment scheme, as reported by Roche^9^ as part of the medicine supply initiative. There is shared responsibility for medication costs, with contributions from the government, pharmaceutical companies, and patients themselves. This approach significantly reduces the economic burden on patients, who are responsible for only 20% of medication costs, thereby easing their financial burden. Oversight of the program rests with the national payer, which manages enrollee registration and hospital onboarding. Participating pharmaceutical companies help ensure product quality and price stability, particularly amid inflationary pressures.

This initiative aligns with the Nigerian Data Protection Act: data remains within national borders and is accessible based on defined roles. Roche, for instance, accesses only anonymized datasets, while NHIA retains access to enrollee-level information. This data has proven instrumental in understanding disease burdens, treatment patterns, and the effect of reimbursement on patient outcomes. Starting with seven pilot centers, the program reimbursed cancer care for >200 patients and generated routine data showing major access barriers (long patient travel distances to the nearest designated treatment center), informing expansion to 22 centers nationwide. Furthermore, patients demonstrated significantly longer adherence to treatment protocols compared to observations in the prior out-of-pocket market, and several patients presented for care at an earlier stage of disease progression: >80% of patients treated under the program were presenting at an early stage of disease^10^, most commonly stage 1 or stage 2. These figures came from program monitoring across participating centers and served as the guiding principles for co-payment adjustments.

By increasing access to healthcare, this partnership not only benefits patients but also facilitates the collection of valuable patient data, which is useful to government bodies and pharmaceutical firms. Further, hospitals and insurance companies gain access to more comprehensive and structured data from a larger number of patients, enabling both operational and clinical improvements in healthcare delivery.

### NGO—research organization collaboration

In Nigeria, partnerships between non-profit foundations and research entities have significantly improved the process of gathering patient data. For instance, the Medicaid Cancer Foundation (a not-for-profit organization) supports cancer patients across the care journey and plays a pivotal role in patient care. They facilitate pre-mammogram consultations and provide radio diagnostic and cancer screening services through their PACE (Patient Access to Care Program)^11^ program that elevates the needs of cancer patients. These services create an important entry point for generating structured patient data and drawing insights on local disease patterns. During consultations, doctors obtain informed consent to collect patient information and notify patients of potential future contact from research organizations (e.g., Yemanja Cancer Care Initiative) dedicated to improving access to early diagnosis and prevention for women’s cancer. Consent and privacy are treated as core pillars during patient interactions.

Such non-profit organizations also use digital tools such as REDCap to collect data from women who have undergone mammography, aiming to compile evidence of treatment effectiveness and advocate for governmental support. Importantly, the foundation’s data collection is directly tied to improving access to care through patient navigation, linking patients to specialists, and facilitating psychosocial support, while also strengthening the evidence base for advocacy, including its annual cancer advocacy program, which draws attention to the plight of cancer patients. These collaborations are particularly valuable because they enable research and data generation within marginalized groups and in contexts that are otherwise challenging to access.

### NGO—healthcare provider collaboration

Non-profit organizations, such as the Clinton Health Access Initiative (CHAI)^12^, can improve healthcare access by supporting medicine availability and strengthening monitoring and evaluation (M&E) systems that generate actionable data for operations and policy. In Nigeria, the Federal Ministry of Health launched the chemotherapy access partnership (CAP)^13^ with CHAI and other partners to expand access to lower-priced, quality-assured chemotherapy medicines through hospital pharmacies in seven teaching hospitals; the program was designed to reduce patients’ costs while stabilizing supply through coordinated ordering and streamlined distribution processes.

Evidence of program scale-up is reported in national assessments: under the Nigeria Cancer Access Partnership Program (NCAP), ~2000 patients had been enrolled across 17 hospitals by the end of 2022^14^, with medicines centrally procured for cost efficiency. The program is also referenced in Nigeria’s national chemotherapy safety policy, which notes that the CAP collaboration has reduced unit costs of drugs in its portfolio, highlighting how partnership design and routine program data can support affordability and system strengthening.

### Digital health company—health provider collaboration

In settings with fragmented health records, digital platforms that connect patients and providers can improve continuity of care while strengthening health-data ecosystems. An example is mDoc, a for-profit digital health company operating across sub-Saharan Africa that connects patients with chronic conditions to providers and offers telehealth support (e.g., self-care coaching and provider networks). Working with government agencies, NGOs, and community groups, mDoc integrates its services into local communities, including through trusted channels such as religious centers that help inform and refer patients. As patients use the app, mDoc collects longitudinal engagement and self-management data that can be used to refine recommendations and generate insights for partners. The company reports 163,000+ members, 16,000+ healthcare workers trained, 2.3 million+ interactions, and 84% of members improving their chronic condition management. These are company-reported figures published on its own website. In addition, independent evidence coming from peer-reviewed evaluations of a hypertension Extension for Community Healthcare Outcomes program co-delivered by mDoc in the Federal Capital Territory reports high acceptability among community health extension workers, measurable knowledge gains, and application of the material in participating primary health centers.

To address governance and trust, mDoc^15^ states that its privacy practices align with Nigeria’s data protection framework (NDPA/NDPR) and that users can review the privacy policy, provide consent, and withdraw consent (opt out). Importantly, mDoc reports that it uses de-identified and aggregated data to train and improve its AI models, does not use identifiable personal data for model training, and applies safeguards to reduce re-identification risk.

### Digital health company—NGO collaboration

Digital decision-support tools can strengthen frontline diagnosis and surveillance for under-recognized conditions, such as neglected tropical diseases (NTDs), while generating structured data for public health action. VisualDx^16^, a web-based clinical decision support system, received a Bill & Melinda Gates Foundation grant^17^ to develop an AI-enabled surveillance and decision-support tool to expand access to accurate diagnosis, referral, and care for NTDs in Africa.

Using AI/ML for image analysis and structured clinical data, the tool supports provider education across varied experience levels and enables tracking and reporting of cases, differentials, and outcomes in Nigeria. Implemented with stakeholders including government agencies, NGOs, and healthcare providers, such collaborations can improve data capture and build longitudinal datasets that support better clinical decisions and public health planning.

On governance, VisualDx describes privacy protections in its privacy policy. Evidence from an implementation in Botswana suggests a data-minimizing approach: VisualDx collects only anonymized/generalized patient information for differential diagnosis, and images used for AI-based analysis remain on-device and are discarded immediately after analysis^18^.

These examples show how well-designed multi-stakeholder collaborations can strengthen health data collection in Nigeria. By making incentives and accountability explicit, they enable governments, NGOs, private companies, and healthcare providers to align around shared goals, while delivering distinct value to each partner (e.g., affordability and coverage for payers; continuity and quality for providers; reach and navigation for NGOs; and operational viability for private partners). Where formal evaluations are not yet available, progress can be monitored through interim indicators such as enrollment and out-of-pocket spending, supply continuity (e.g., stock-outs), data governance and quality (e.g., consent rates, completeness, role-based access), and evidence of learning (e.g., program adjustments based on routine monitoring). Collectively, these elements underscore the role of structured partnerships in bridging healthcare gaps and generating actionable data to improve outcomes and inform policy.

At the same time, these examples should be understood as promising configurations to be tested and adapted rather than as universally proven models. Evidence on health-related public-private partnerships in low- and middle-income countries remains mixed, with reported benefits in service quality and cost management alongside persistent concerns about equity and sustainability. A recent systematic review identifies five factors that appear particularly important for effective partnerships in low-income settings: leadership support and alignment with broader priorities, local ownership and frontline engagement, shared learning across teams, resilience in responding to external challenges, and robust data systems to monitor progress^19^. These conditions provide a useful lens for understanding enabling conditions that can foster transferality.

## Recommendations for other developing countries

The integration of advanced data analytics and AI into healthcare in Nigeria offers valuable insights for other developing countries seeking to address healthcare gaps through collaborative public-private partnerships. Drawing from Nigerian cases and our multi-year collaboration, we propose the following recommendations:

### Prioritize data diversification and representation

Healthcare stakeholders must focus on collecting and utilizing healthcare data from diverse and underrepresented populations. This approach is essential for mitigating biases in AI models, improving their generalizability, and ensuring equitable healthcare solutions. For example, collecting data from rural, indigenous, or marginalized communities and incorporating these insights into AI tools will help reduce disparities and ensure that solutions cater to the broader spectrum of healthcare demands. Because partnership-based data collection tends to follow insurance coverage and tertiary facilities, representation targets should be set explicitly at the outset and monitored, rather than assumed to follow from expanded access.

### Foster strategic public-private partnerships

Countries with limited resources can emulate Nigeria’s model of forming strategic partnerships among governments, pharmaceutical companies, research institutions, patient advocacy groups, and insurance companies. Such collaborations allow stakeholders to align public health interests with corporate goals. Governments can facilitate infrastructure development, improve access to healthcare data, ensure regulatory compliance, and avoid data silos. Pharmaceutical companies can contribute by providing funding, technology, and expertise while benefiting from insights into disease trends and treatment outcomes. Non-profit organizations and research institutions can bridge gaps by working directly with communities to collect and analyze critical healthcare data. These partnerships create synergy between public welfare and private enterprise, enabling innovations that address data scarcity and improve health outcomes. Governments entering such arrangements should negotiate exit, custodianship and continuity terms at the point of agreement, when their leverage is greatest, rather than after dependency has formed.

### Engage local communities

Community involvement is crucial for the success of collaborative frameworks. Leveraging trusted local institutions, such as religious centers, schools, and community organizations helps build trust and encourages participation in healthcare initiatives. For example, in Asia, partnerships with religious organizations or village councils can enhance community buy-in for data collection efforts. Engaging communities also generates richer datasets that reflect the unique health experiences of diverse populations, ensuring that AI-driven healthcare solutions are well-aligned with local needs.

### Focus on policy innovation and sustainable infrastructure

Policymakers in developing countries must draw on the lessons from Nigerian collaborative frameworks to create long-term solutions. Because Nigeria lacks centralized EHRs and data infrastructures remain fragmented, many initiatives strengthen data capture within specific care and financing workflows rather than replacing national health information systems. This can improve data quality and service delivery, but it also risks creating new project-specific data silos if interoperability and public-sector access pathways are not planned for. Key strategies, therefore, include enacting policies that incentivize data sharing and collaboration; investing in enabling infrastructure (e.g., EHR systems, shared minimum datasets/standards, and broadband internet) to support interoperable data collection and analysis; and ensuring that healthcare data innovation is aligned with national health goals, equity considerations, and secure reuse of de-identified outputs for health system planning.

## Adopt a commitment to inclusivity and sustainability

The success of collaborative frameworks depends on a shared commitment to inclusivity, community engagement, and sustainable approaches. African nations have demonstrated the value of aligning stakeholder goals through accountability and incentive-based models. By replicating these principles, other developing countries can create scalable and impactful healthcare frameworks.

### Build an independent evaluation from the outset

Each of the partnerships described here generates data about itself, and in most cases, that data is held and reported by the partners with an interest in the result. Countries adopting similar arrangements should require, as a condition of partnership, that routine program data be accessible to independent researchers under controlled-access terms, and that evaluation questions and indicators be fixed before the program begins. This is inexpensive at the design stage and close to impossible to retrofit.

Taken together, the applicability of these recommendations in other settings depends on enabling conditions such as an enforceable data-protection framework, a functioning payer or financing mechanism, sufficient market scale to make it attractive for the private sector, trusted civil-society intermediaries, and adequate clinical capacity. These conditions cannot be assumed elsewhere, and countries with weaker regulatory, financing, or health-system infrastructures may require additional safeguards and adaptation. Transfer also requires attention to who is represented in the resulting data: in Nigeria, many initiatives disproportionately capture insured, urban, and tertiary-care populations, while rural and out-of-pocket populations remain less visible. Countries adopting similar approaches should therefore plan explicitly for the inclusion of underserved populations in both care pathways and data ecosystems, or risk reproducing the selection biases these partnerships seek to address.

In addition, public-private partnerships are not neutral instruments; each arrangement creates risks, dependencies, and private-sector incentives shape what is funded, measured, and sustained. Heavy reliance on a single commercial actor can expose services and data infrastructures to operational or financial disruption. Partnerships should therefore define in advance how services and data will be maintained or transferred if a partner withdraws, including clear arrangements for data access, custodianship, transition responsibilities, and continuity financing^20^. Partnership-generated data may also reflect commercial priorities, producing richer datasets for diseases and populations aligned with market incentives while leaving less commercially attractive areas underrepresented, which risks reproducing existing selection biases^21^. In addition, when African health data are used by commercial or international partners, analytical assets and other benefits may not necessarily return to the institutions and communities that generated them. Safeguards such as controlled-access environments, benefit-sharing agreements, custodianship arrangements, and licensing terms that define permitted uses and returns should therefore be built into partnerships from the outset^22^.

## Concluding remarks

Nigeria’s emerging partnership ecosystem shows that closing the health-data gap is possible even in fragmented, resource-constrained settings. When incentives are aligned, trust is earned, and data governance is treated as foundational rather than an afterthought. These remain promising configurations rather than validated models, and we have been explicit about where the supporting evidence is strong and where it is partner-reported. Treating them as a starting point for evaluation, rather than as a template for replication, is what would allow the next country to learn more from Nigeria’s experience than Nigeria has yet been able to learn from its own. The next step is to move from promising pilots to durable, interoperable infrastructures that protect rights, reward participation, and keep value creation local: shared minimum datasets and standards, clear consent and access pathways, independent evaluation arrangements agreed in advance, and mechanisms that return benefits to communities and frontline providers. If developing countries invest in these building blocks now, they can avoid importing inequitable AI and instead shape AI-enabled care that is accurate, accountable, and genuinely inclusive: turning today’s data scarcity into tomorrow’s opportunity for more equitable health outcomes.

## Supplementary information

Transparent Peer Review file

## Data Availability

The datasets generated and/or analyzed during the current study are not publicly available due to the protection of the privacy of participants, but are available from the corresponding author on reasonable request.
