# Supplementary material for: Learning from Nigeria’s public-private pathways to generate inclusive healthcare data
Source: Commun Med (Lond). 2026 Oct 21;6:548. doi: 10.1038/s43856-026-01984-x (PMC13687552; doi:10.1038/s43856-026-01984-x)
Supplement: Supplementary file 1 [file 43856_2026_1984_MOESM1_ESM.pdf]

nature portfolio

Peer Review File

Learnings from Nigeria's Public-Private Pathways to Generate Inclusive Healthcare Data

Corresponding Author: Professor Yash Shrestha

This file contains all reviewer reports in order by version, followed by all author rebuttals in order by version.

Version 0:

Reviewer comments:

Reviewer #1

(Remarks to the Author)
Summary/Overall impression of work

This comment addresses two very relevant topics in healthcare: the risk of biased data sets and digital tools, and the role of public-private partnerships in developing countries. It provides a useful overview of concrete PPP collaborations in Nigeria. However, the paper does not yet provide sufficient information on project oversight, data governance or evidence for success in the examples. Without this information, it is difficult to evaluate the projects and judge whether they could serve as a blueprint for other developing countries.

Major comments
1. In the introduction and throughout the paper, statements lack supporting references (e.g., AI opportunities, healthcare and data scarcity challenges in Nigeria, digital divide).

2. The paper lists concrete and interesting examples of different PPP. However, the project descriptions provide only limited or no details on project oversight, roles and responsibilities, data governance frameworks (including sharing, access, permitted use cases, safety and security measures especially with cross-boarder data transfer), incentive models or public engagement strategies to overcome structural inequities. Only one project mentioned informed consent. While the manuscript mentions that "African nations have demonstrated the value of aligning stakeholder goals through accountability and incentive-based models", it misses to provide the data and necessary details from the presented projects on this statement.

3. The paper would be strengthened by clearer presentation of evidence and success indicators of the projects. At present, it is unclear how project success is defined for the various project partners, what are the key performance measures and how are they measured, what is the evaluation plan and whether any project outcomes have been formally reported or published. Since the manuscript suggests these projects as blueprints for other developing countries, documenting concrete measures of effectiveness is essential.

4. The suggestion of the manuscript that the projects can serve as blueprints for other developing countries is promising but currently underdeveloped with regards to necessary enabling infrastructures, resources and governance frameworks. What are the specific conditions making the projects a success in Nigeria? Could there be possible constraints in other developing countries?

5. The paper would benefit from a critical reflection on risks of collaborations as they are described in the manuscript, such as long-term dependencies of governments on private partners or ethical concerns of exploitations if treatments are only available for those who share data. And more specifically, how are those risks addressed in the mentioned projects making them truly blueprint worthy.

6. The authors identify absence of centralised EHR systems and fragmented data infrastructures as issues for care delivery. It would be helpful to explain how the mentioned projects contribute to overcoming these issues or if they risk creating further data silos. Do they integrate with National Health Data Initiatives or does data access mainly remain with the project partners? Clarification here would be valuable to assess the potential long-term impact as well as sustainability of the

projects.

Minor comments
A brief abstract would be very helpful.
Wrong punctuation in line 11

Version 1:

Reviewer comments:

Reviewer #1

(Remarks to the Author)
The manuscript greatly improved from the revision. However, to serve as an actual blueprint, evidence and transferability conditions are still missing. Especially without evidence (published studies, case studies, etc) it's a promising example rather than a blueprint. A critical reflection on risks such as private sector dependencies and commercial incentives is missing.

Open Access This Peer Review File is licensed under a Creative Commons Attribution 4.0 International License, which permits use, sharing, adaptation, distribution and reproduction in any medium or format, as long as you give appropriate credit to the original author(s) and the source, provide a link to the Creative Commons license, and indicate if changes were made.
In cases where reviewers are anonymous, credit should be given to 'Anonymous Referee' and the source.
The images or other third party material in this Peer Review File are included in the article's Creative Commons license, unless indicated otherwise in a credit line to the material. If material is not included in the article's Creative Commons license and your intended use is not permitted by statutory regulation or exceeds the permitted use, you will need to obtain permission directly from the copyright holder.
To view a copy of this license, visit https://creativecommons.org/licenses/by/4.0/

Summary/Overall impression of work<br><br>This comment addresses two very relevant topics in healthcare: the risk of biased data sets and digital tools, and the role of public-private partnerships in developing countries. It provides a useful overview of concrete PPP collaborations in Nigeria.<br><br>However, the paper does not yet provide sufficient information on project oversight, data governance or evidence for success in the examples. Without this information, it is difficult to evaluate the projects and judge whether they could serve as a blueprint for other developing countries.	We would like to thank the reviewer for their thoughtful comments and suggestions. Within the space limitations, we collected more data which allowed us to add further details to the examples. We hope the updated manuscript will provide sufficient evidence and showcase the repeatability of the projects mentioned.
2. The paper lists concrete and interesting examples of different PPP. However, the project descriptions provide only limited or no details on project oversight, roles and responsibilities, data governance frameworks (including sharing, access, permitted use cases, safety and security measures especially with cross-border data transfer), incentive models or public engagement strategies to overcome structural inequities. Only one project mentioned informed consent. While the manuscript mentions that “African nations have demonstrated the value of aligning stakeholder goals through accountability and incentive-based models”, it misses to provide the data and necessary details from the presented projects on this statement.	Thank you for this important suggestion. We strengthened the partnership descriptions by adding concrete details on oversight, roles and responsibilities, data governance, incentive models, and public engagement/consent practices where relevant information was available and accessible. For example, for the NHIA–pharmaceutical co-payment initiative, we now specify the national payer’s oversight role (enrollment and hospital onboarding), the responsibilities of participating pharmaceutical companies (price stability/product quality), and a clear data governance approach (role-based access, anonymized access for industry partners, and in-country data handling aligned with Nigeria’s data protection requirements). We made similar improvements to all PPP examples.
	Thank you for your critical insight. We agree and have made the manuscript more evidence-based by adding success indicators and reported outcomes where available. For the NHIA–pharmaceutical co-payment pilot, we report program reach (200+ patients treated in the pilot phase) and describe how pilot data drove concrete operational changes (expansion of treatment centers) etc. For the Chemotherapy Access Partnership, we added reported scale-up metrics (enrollment of ~2,000 patients across 17 hospitals by end-2022; expansion beyond the pilot). For the

	VisualDx initiative, we strengthened evidentiary support by citing grant documentation and an African case study reporting privacy-preserving, data-minimizing features. We also clarify that “success” can differ by partner (access/affordability, continuity of supply, quality assurance, data completeness, patient trust and consent, and use of data for decision-making), and we propose pragmatic indicators for replication when formal evaluations are pending.
4. The suggestion of the manuscript that the projects can serve as blueprints for other developing countries is promising but currently underdeveloped with regards to necessary enabling infrastructures, resources and governance frameworks. What are the specific conditions making the projects a success in Nigeria? Could there be possible constraints in other developing countries?	Thank you for highlighting the importance of generalizability of our suggestions. The sustainability and mitigation of risks are heavily reliant on political will. It is essential for the longevity of these initiatives that the partnerships are strengthened and, crucially, institutionalized. In many of the partnerships discussed, significant efforts have been made to enshrine these programs within government frameworks. For instance, several programs have been integrated into national budget lines and included in the national essential drug lists. This institutionalization is a deliberate strategy to ensure the programs survive potential regime and management changes and maintain long-term viability. It is important to also add that the government recognizes the vital role that the private sector plays in the development of the healthcare landscape.
5. The paper would benefit from a critical reflection on risks of collaborations as they are described in the manuscript, such as long-term dependencies of governments on private partners or ethical concerns of exploitations if treatments are only available for those who share data. And more specifically, how are those risks addressed in the mentioned projects making them truly blueprint worthy.	Thank you for highlighting the valid concern around risks of collaborations, especially concerning the risk of creating further data silos. A key mitigating factor, which we must highlight, is that the government entities involved in these public-private partnerships have been granted full access to the data. They also serve as formal data custodians. This arrangement ensures that when a national Electronic Health Record (EHR) system is fully implemented and operational, the data can be seamlessly transferred and integrated, thus preventing permanent fragmentation.
6. The authors identify absence of centralised EHR systems and fragmented data infrastructures as issues for care delivery. It would be helpful to explain how the mentioned projects contribute to overcoming these issues or if they risk creating further data silos. Do they integrate with National Health Data Initiatives, or does data access mainly remain with the project partners? Clarification here would be valuable to assess the potential long-term impact as well as sustainability of the	Thank you. We revised the manuscript to clarify how the described initiatives interact with Nigeria’s fragmented data landscape and to acknowledge the risk of creating additional data silos.<br><br>W<br>strengthen routine data capture within existing care pathways rather than building standalone datasets. For example, the NHIA–pharmaceutical co-payment

projects.	We also strengthened the Recommendations section to address sustainability and the risk of new data silos. We now state that, because centralized EHRs are lacking and data infrastructures are fragmented, many initiatives improve data capture within specific workflows but may create project-specific silos if interoperability and public-sector access pathways are not planned. Accordingly, we recommend investing in enabling infrastructure (e.g., EHR systems and broadband) and aligning healthcare data innovation with national health goals and equity considerations to avoid reinforcing fragmentation.”
A brief abstract would be very helpful.<br>Wrong punctuation in line 11	Thank you. A brief abstract has been added, and the punctuation mistakes have been identified and corrected.

"The manuscript greatly improved from the revision. However, to serve as an actual blueprint, **evidence** and **transferability conditions** are still missing. Especially without evidence (published studies, case studies, etc) it's a promising example rather than a blueprint. A **critical reflection** on risks such as private sector dependencies and commercial incentives is missing."	We thank the reviewer for the positive assessment and for the three remaining points, which we have addressed as follows.<br><br>**Evidence:** We have strengthened the empirical basis of the manuscript and reframed claims that rested on self-reported data. Company-reported metrics are now explicitly attributed as such: the mDoc figures are identified in the text as figures published by the company on its own website. Against these, we have added independent peer-reviewed evidence: published evaluations of a hypertension ECHO program co-delivered by mDoc with community health extension workers in the Federal Capital Territory, reporting acceptability, knowledge gains and use of the material in participating primary health centres (Baldridge et al. 2025). For the NHIA co-payment scheme we now report the source of the figures, stating that stage-at-presentation and enrolment data came from programme monitoring across participating centres, and we have updated the expansion figure to 22 centres nationwide. We have also added a paragraph situating the Nigerian cases within the wider literature, noting that evidence on health public–private partnerships in low- and middle-income countries is mixed, and drawing on a systematic review of 144 studies that identifies five factors associated with effective partnerships in low-income settings (Iroz et al., 2024).<br><br>**Transferability conditions:** We agree that a blueprint without stated conditions is not a blueprint, and we have accordingly revised the claim and specified the conditions. The preface and introduction no longer describe the Nigerian experience as a replicable blueprint but as a set of transferable design principles whose transfer depends on stated conditions; the recommendations section has been revised in the same terms. A new passage now names the enabling conditions we consider necessary, an enforceable data-protection framework, a functioning payer or financing mechanism, sufficient market scale to attract private investment, trusted civil-society intermediaries and adequate clinical capacity, and states that these cannot be assumed elsewhere. We also now acknowledge a limit on our own cases: initiatives in Nigeria disproportionately capture insured, urban and tertiary-

	care populations, and countries adopting similar approaches must plan explicitly for including underserved populations or risk reproducing the selection biases these partnerships are meant to address.<br><br>**Critical reflection on risks** We have added a new passage stating directly that public–private partnerships are not neutral instruments. It addresses three risks. First, dependency: heavy reliance on a single commercial actor exposes services and data infrastructures to operational or financial disruption, and partnerships should therefore define in advance how services and data are maintained or transferred if a partner withdraws, including data access, custodianship, transition responsibilities and continuity financing (Hellowell, 2019). Second, incentive-shaped data: partnership-generated data may reflect commercial priorities, producing richer datasets where market incentives are strongest and leaving less commercially attractive areas underrepresented (Abebe et al., 2021). Third, value capture: where African health data are used by commercial or international partners, the resulting analytical assets may not return to the institutions and communities that generated them, and we recommend controlled-access environments, benefit-sharing agreements, custodianship arrangements and licensing terms defining permitted uses and returns as terms of the partnership from the outset (Munung et al., 2025).
